# Association between Embolic Stroke Patterns, ESUS Etiology, and New Diagnosis of Atrial Fibrillation: A Secondary Data Analysis of the Find-AF Trial

**DOI:** 10.1155/2017/1391843

**Published:** 2017-04-27

**Authors:** Ilko L. Maier, Katharina Schregel, André Karch, Mark Weber-Krueger, Rafael T. Mikolajczyk, Raoul Stahrenberg, Klaus Gröschel, Mathias Bähr, Michael Knauth, Marios-Nikos Psychogios, Rolf Wachter, Jan Liman

**Affiliations:** ^1^Department of Neurology, University Medicine Göttingen, Göttingen, Germany; ^2^Department of Neuroradiology, University Medicine Göttingen, Göttingen, Germany; ^3^Research Group Epidemiological and Statistical Methods (ESME), Department of Epidemiology, Helmholtz Centre for Infection Research, Braunschweig, Germany; ^4^Department of Cardiology and Pneumology, University Medicine Göttingen, Göttingen, Germany; ^5^Department of Neurology, University Medicine Mainz, Mainz, Germany

## Abstract

*Background*. Atrial fibrillation (AF) is an important cause of embolic stroke of undetermined source (ESUS). Imaging-patterns like multiple infarcts, simultaneous involvement of different circulations, infarcts of different ages, and isolated cortical infarcts are likely to indicate cardioembolic stroke. The aim of our study was to evaluate the association between embolic stroke patterns, ESUS, and the new diagnosis of AF.* Methods*. Stroke etiology and imaging characteristics from patients included in the Find-AF study were obtained. Embolic stroke patterns in CT- or MR-imaging were correlated with the diagnosis of ESUS as well as the short- (on baseline ECG and during 7-day Holter) and long-term (12-month follow-up) diagnosis of AF.* Results*. From 281 patients included in the Find-AF study, 127 (45.2%) patients with ischemic lesions detected in CT or MRI were included. 26 (20.5%) of these patients had ESUS. At least one embolic stroke pattern was detected in 67 (52.7%) patients. Embolic stroke patterns were not associated with ESUS (OR 1.57, 0.65–3.79, *p* = 0.317), the short-term (OR 0.64, 0.26–1.58, *p* = 0.327) or long-term diagnosis of AF (OR 0.72, 0.31–1.68, *p* = 0.448).* Conclusions*. This secondary data analysis of the Find-AF study could not provide evidence for an association between embolic stroke patterns, ESUS, and the new diagnosis of AF.

## 1. Introduction

Early diagnosis of stroke etiology is crucial for secondary prevention in patients with acute ischemic stroke. In cardioembolic stroke, risk of recurrence is highest in the first weeks after stroke (around 10%) and drops to 5% in the following 12 months [1]. There are effective strategies in secondary prevention depending on the diagnosed stroke subtype. For patients with a definite cardioembolic source, secondary prevention with vitamin K antagonists or new oral anticoagulants (NOACs) reduces the risk for stroke recurrence by 60–70% [2].

About 25% of all ischemic strokes are classified as cryptogenic, of which the majority is likely to be of embolic origin, including major- (like atrial fibrillation or left ventricular thrombi) and minor-risk (like ventricular dysfunction, mitral annular calcification, or patent foramen ovale) cardiac sources [3–6]. It has been suggested that a high proportion of cryptogenic ischemic strokes are caused by asymptomatic paroxysmal atrial fibrillation (AF). In this context studies following two approaches have been initiated recently: first, optimization of ECG-monitoring with extended Holter recording [7, 8] and, second, treatment of patients with the newly proposed concept of an “embolic stroke of undetermined source (ESUS)” with NOACs without detection and diagnosis of atrial fibrillation, for example, in the upcoming RE-SPECT ESUS, NAVIGATE ESUS, or ATTICUS trials [9]. The ESUS-term hereby applies to all patients without the diagnosis of a definite cardiac source and without evidence for large or small vessel disease.

Aim of our study is to identify imaging parameters which are associated with ESUS and the new diagnosis of AF in acute stroke patients to find another approach of identifying patients with embolic stroke which are likely to benefit from oral anticoagulation even in the absence of a definite diagnosis of an embolic source.

## 2. Materials and Methods

### 2.1. Patient Population and Study Design

In this secondary data analysis, we evaluated clinical and imaging data from the monocentric, observational Find-AF trial (ISRCTN 46104198). Details of the Find-AF trial have been published previously [10]. In brief, patients with acute stroke or transient ischemic attack (TIA) and sinus rhythm at admission were enrolled. Screening for AF was performed with 7-day Holter. In addition, a new diagnosis of AF was obtained on 12-month follow-up examinations (clinical assessment and 12-lead-ECG). AF was defined as any atrial arrhythmia lasting >30 seconds. The protocol of the Find-AF trial was approved by the ethics committee of the University Medicine Göttingen and all patients gave written informed consent.

The diagnosis of ESUS applied to patients with nonlacunar stroke (defined as subcortical infarcts ≤15 mm in cCT- or ≤20 mm DWI-MR-imaging caused by small vessel occlusion), absence of extra- or intracranial ≥ 50% luminal stenosis proximal to the infarct, major-risk cardioembolic source of embolism (including the absence of atrial fibrillation in the first 24 h of the 7 d Holter), and other specific causes of stroke [9] (see the following list).

*Diagnostic Criteria for an Embolic Stroke of Undetermined Source (ESUS; [9]). *Exclusion of the following:

- * Intra- or extracranial stenosis* ≥50% (North American Symptomatic Carotid Endarterectomy Trial- (NASCET-) criteria) or occlusions supplying the area of ischemia
- * Evidence of cardioembolic pathologies* (major cardiac sources)
  - * Exclusion of atrial fibrillation*: No history of atrial fibrillation, atrial fibrillation on baseline ECG or in the first 24 h of the 7 d Holter
  - No evidence for sick sinus syndrome, atrial asystole, cardiac thrombus, mechanic valve replacement, myxoma or cardiac tumor, mitral valve stenosis, recent myocardial infarction (<4 weeks) or heart failure (EF < 30%), and endocarditis
- * Lacunar infarct pattern*  caused by small vessel occlusion in white matter (<15 mm cCT, <20 mm DWI-MR)
- * No evidence for rare stroke etiologies* like: migrainous infarction, vasculitis, or thrombophilia

In the analysis for the association between stroke patterns and new diagnosis of AF and ESUS, only patients with a detection of acute or subacute stroke in CT or MRI attributable to initial clinical symptoms were included. Patients with a history of AF prior to index stroke have been excluded. Patients with new AF on baseline ECG or new AF in 7 d Holter were considered as short-term-AF-positive. Patients with the new diagnosis of AF between baseline ECG and 12-month follow-up were considered as long-term-AF-positive.

### 2.2. Imaging

Embolic stroke patterns were retrospectively evaluated on CT- and MR-imaging performed on admission or during inpatient stay after index ischemic stroke. Every patient received CT-imaging. In patients with additional MR-imaging, MR data were evaluated instead of CT-imaging. The senior neuroradiologist classifying the embolic stroke patterns was unaware of the clinical status of the included patients.

Stroke patterns were defined as multiple acute infarcts, simultaneous involvement of different circulations, multiple infarcts of different ages, and isolated cortical ischemia (see the following list).

*Imaging Criteria for Embolic Stroke Patterns [11]*

- * Multiple acute infarcts:* MRI: multiple noncontiguous lesions, which were hyperintense on DWI and hypointense on ADC maps; CT: multiple, noncontiguous, and hypodense lesions
- * Simultaneous involvement of different circulations:* MRI/CT: multiple acute ischemic lesions in both right and left anterior circulations or in both anterior and posterior circulations
- * Multiple infarcts of different ages:* MRI: ischemic lesions with hyperintense signals on DWI that meet 2 of the following 3 criteria: hypointense on ADC and isointense on FLAIR (hyperacute); hypointense on ADC and hyperintense on FLAIR (early acute); isointense on ADC and hyperintense on FLAIR (late acute or subacute); CT: simultaneous presence of acute, subacute, and/or old (liquorisodense) ischemic lesions
- * Isolated cortical ischemia:* MRI/CT: presence of multiple, isolated cortical ischemic lesions

The presence of microangiopathy was defined as either punctual or confluent white matter T2w-hyperintensities in MRI or hypodensities on CT. The extent of microangiopathic changes in stroke imaging was quantified using the Fazekas scale for MRI and adapting it for CT by evaluating hypodense white matter lesions. The score ranged from 0 (no microangiopathic changes) to 3 (large, confluent microangiopathic changes) [12].

### 2.3. Statistical Analyses

Baseline characteristics were assessed using relative frequencies and means (±standard deviations). Groups were compared using chi-squared tests and univariable logistic regression models. All data analyses were performed using Stata 12 (College Station, USA).

## 3. Results

**Figure 1:**
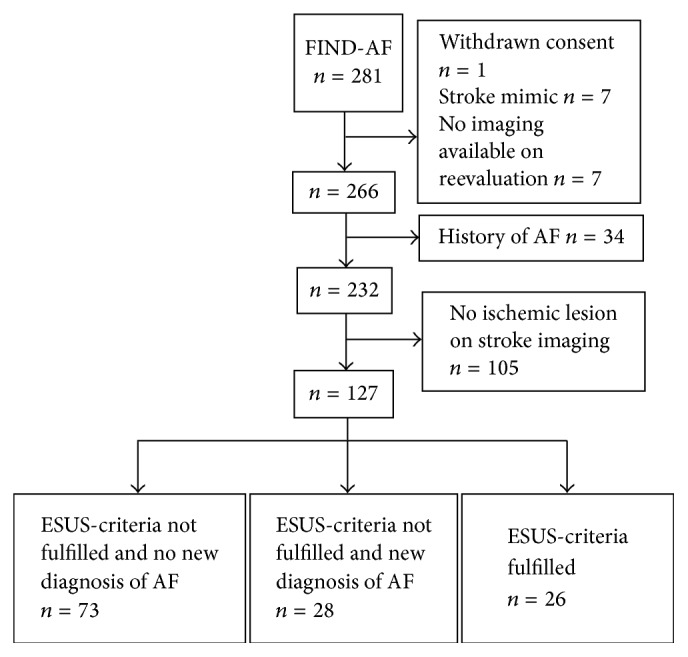
Study flow chart of included patients in this imaging substudy of the Find-AF trial.

From 281 patients included in the Find-AF study, 127 patients were eligible for this imaging substudy. As shown in in the flow-diagram in Figure 1, 34 patients with a history of AF and 105 patients without ischemic lesions on cerebral imaging were excluded. As shown in Table 1, baseline characteristics did not differ between groups with the exception of a higher number of patients with a history of coronary artery disease in the group of patients with the new diagnosis of AF (*p* = 0.019). Of the 127 patients included, 65 (51.2%) had baseline MRI. Of the 105 excluded patients, MR-imaging was performed in 32 (30.5%). At least one embolic stroke pattern was detected in 67 (52.6%) patients.

The most frequent reasons for nonfulfillment of the ESUS-criteria were lacunar strokes and a stenosis of ≥50% according to the North American Symptomatic Carotid Endarterectomy Trial- (NASCET-) criteria (Supplementary Table 1, Supplementary Material available online at https://doi.org/10.1155/2017/1391843). 26 patients (20.5%) fulfilled the criteria for ESUS. There was a higher number of patients with multiple infarcts of different ages on the ESUS-group, which did not reach statistical significance (42% versus 26%; *p* = 0.102). There was no association between other stroke patterns or the presence of at least one embolic stroke pattern and ESUS etiology (OR 1.57, 0.65–3.79, *p* = 0.317; Table 2). The rate of new diagnosis of AF between baseline and 12-month follow-up was lower in the ESUS-group compared to the non-ESUS-group (4/26 (15.4%) versus 28/101 (27.7%)).

New AF was diagnosed in 23 (18.1%) patients in baseline ECG and/or 7-day Holter and in additional 5 (3.9%) patients in the period between 7-day Holter and 12-month follow-up. Embolic stroke patterns in general as well as the prevalence of at least one embolic stroke pattern were neither associated with the short-term nor with the long-term diagnosis of AF (Tables 3 and 4). Including patients with a history of AF in the analysis also revealed no association between embolic stroke patterns and AF (data not shown).

As shown in Supplementary Tables 2 and 3, we performed an analysis in the subgroup of patients with MRI. This subanalysis revealed a trend towards a higher number of patients with multiple infarcts of different ages (54% versus 26%, OR 3.26, 0.86–12.39, *p* = 0.083) and isolated cortical infarctions (27% versus 7%, OR 4.50, 0.84–23.99, *p* = 0.078) in the ESUS-group (Supplementary Table 2). As in the analysis of the whole study group, embolic stroke patterns were not predictive for the new diagnosis of AF on baseline ECG or 7-day Holter (Supplementary Table 3).

Neither the presence nor the extent of microangiopathy was associated with ESUS or the new diagnosis of AF. Microangiopathy was present in 80% of patients with versus 79% of patients without ESUS (*p* = 1.000) and in 91% of patients with versus 77% without the new diagnosis of AF (*p* = 0.158). The extent of microangiopathic changes was not different in patients with and without ESUS or with and without the new diagnosis of AF (median Fazekas score was 1 in all groups (IQR, 1-2; *p* = 0.402 and *p* = 0.370, resp.)).

## 4. Discussion

In the present study, we found no evidence for an association between embolic stoke patterns and the new diagnosis of AF or ESUS etiology in patients included in a prospective trial on the new diagnosis of AF after stroke with extended Holter monitoring.

A similar approach was made in a recent subgroup analysis of the CRYSTAL-AF study, in which infarct topography of patients with cryptogenic stroke and the new diagnosis of AF by an insertable cardiac monitor have been investigated [13]. In that study, the authors did not find any acute stroke pattern to be associated with AF detection; an association was found only for chronic brain infarctions and leukoaraiosis. Concerning these imaging characteristics, our study similarly was not able to identify an acute stroke pattern associated with the new diagnosis of AF. The number of patients with old ischemia and microangiopathy was higher in the group with the new diagnosis of AF (91% versus 77%), which, however, did not reach statistical significance. As the comorbidity with cardiovascular risk factors is high in stroke patients investigated in both the CRYSTAL-AF study and our subgroup analysis, it seems unlikely that the presence of microangiopathic changes is of great use to be a reliable predictor for AF. In contrast, the lack of statistical significance in our study could indicate a problem of statistical power, as the overall sample size was higher in the CRYSTAL-AF substudy (*n* = 212) compared to our study (*n* = 127).

In our study, detection rates of AF after 12 months were higher compared to the detection rate of the CRYSTAL-AF study (Find-AF subgroup 28 (22.0%) patients; CRYSTAL-AF 12.4%). On the one hand, this higher detection rate would have made it more likely to find an association between stroke patterns and AF; on the other hand, 75.1% of patients included in the CRYSTAL-AF substudy had cMRI versus 51.2% of patients in our analysis. The higher proportion of CT-based assessment of stroke patterns makes it more likely to miss (embolic) infarcts.

Despite the differences in study design, numbers of MRI, and included patients, our results go in line with the findings of the CRYSTAL-AF substudy and cast doubt of the clinical value of infarct topography in cryptogenic stroke and ESUS. The lack of an association between stroke patterns and embolic stroke etiologies in both our and the CRYSTAL-AF study contradicts mechanistic considerations, because it seems likely that underlying stroke etiology in a high proportion of patients with cryptogenic stroke or ESUS is AF and that these patients are likely to show embolic stroke patterns in brain imaging caused by embolization in different circulations or shattered emboli. Moreover, the subpopulation of stroke patients with cryptogenic stroke and AF has an increased risk of recurrence [9]. The risk of recurrent ischemic stroke has again been associated with multiple acute infarcts, simultaneous involvement of different circulations, multiple infarcts of different ages, isolated cortical ischemia, and clinically silent ischemia on MR-imaging [11, 14, 15]. A reason for the missing association between imaging and stroke etiology might be based on the efficacy of cardiac monitoring (CRYSTAL-AF trial) or length of monitoring (Find-AF trial). In this respect, Poli et al. were able to show that rhythm monitoring by a new event recorder (Medtronic Linq instead of XT device) and preselection of patients by the means of echocardiographic or indirect electrophysiological signs for underlying AF in cryptogenic stroke patients can be much more efficient with detection rates of AF up to 31% after 6 months of monitoring [16]. Therefore, the proportion of patients with ESUS and AF might be underestimated; AF patients could have been misclassified to the non-AF group in our study and the results of the association analyses would have been biased towards unity.

Another reason for a lack of association between stroke patterns and AF in both the Find-AF and the CRYSTAL-AF study could be the inclusion of patients with small strokes and low National Institutes of Health Stroke Scale (NIHSS) (median NIHSS in the CRYSTAL-AF was <2 and in our study 3), as there is a known association between large infarct volumes and high NIHSS in patients with cardioembolic strokes caused by AF [17]. Recently, the characteristics of ESUS patients have been described in the Athens Stroke registry (275 patients with ESUS), in which the overall median NIHSS was 5, whereas median NIHSS in patients with diagnosed cardioembolic stroke was 13. Besides the problem of etiologic attribution of the index stroke to poststroke detection of AF, ESUS etiology was not associated with the new diagnosis of AF in our study. Given the low median NIHSS in the Find-AF study, competing causes of embolic stroke patterns like ruptured plaques without hemodynamic relevance, minor cardioembolic sources, or embolic plaques in the aortic arch could have caused the most minor strokes in the ESUS-group compared to other study populations. This assumption is supported by the fact that rates of new diagnosis of AF were lower in the ESUS-group compared to patients not fulfilling the ESUS-criteria. In this respect, our study could not provide evidence for the additional value of imaging characteristics in patient selection for the oral anticoagulation in ESUS-cases.

The strength of our study is the high data quality and integrity of the prospectively designed Find-AF study and the blinded assessment of stroke imaging by an experienced senior neuroradiologist. The major limitation of our study is the low number of patients with detectable ischemic lesions attributed to the index stroke symptoms and the low number of patients with MRI, which is more likely to detect (small) embolic strokes compared to CT-imaging. It is likely that if more patients without ischemic lesions in the baseline CT would have received MRI, the statistical power of our subgroup analysis would have been higher, as the rate of TIA-patients with ischemic lesions on MRI has been described to be higher than 30% [18]. Moreover, a subgroup analysis of patients with MRI revealed a trend towards a higher number of ischemic lesions of different ages and isolated cortical infarcts in ESUS patients (Supplementary Table 2). These findings, however, must be interpreted with caution because of small patient numbers in the different groups and large confidence intervals. In conclusion, these limitations stress the importance to use MRI in future studies investigating the predictive value of embolic stroke patterns on AF or ESUS as stroke etiology.

## 5. Conclusions

Neither our data nor the subgroup analysis of the CRYSTAL-AF study could find convincing evidence for an association between ischemic stroke patterns in acute brain imaging and the new diagnosis of AF. In addition, our study could not provide evidence for a predictive value of stroke imaging for the diagnosis of ESUS. It will be important for future studies to further investigate the link between ischemic stroke patterns with larger sample sizes including patients with major stroke and MR-based stroke imaging as well as investigating patients receiving more sensitive devices (e.g., Linq) or prolonged monitoring with Holter ECG, like in the Find-AF randomized study [19].

## Supplementary Material

The supplementary material includes: additional information about the ESUS-group (reasons for non-fulfillment of the ESUS-criteria) and findings in the subgroup with MR-imaging (association between embolic stroke patterns, ESUS and AF).

## Figures and Tables

**Table 1:** Baseline characteristics (*n* = 127).

	No-ESUS and no new AF (*n* = 73)	New AF (*n* = 28)	ESUS (*n* = 26)	*p* value^*∗*^
Age (years, IQR)	70 (60–78)	77 (69–82)	74 (58–79)	0.137
Sex (male, %)	48 (65.8)	13 (46.4)	11 (42.3)	0.157
Arterial hypertension (*n*, %)	57 (78.1)	18 (64.3)	17 (65.4)	0.816
Diabetes mellitus (*n*, %)	19 (26)	9 (32.1)	6 (23.1)	0.434
Hyperlipidemia (*n*, %)	24 (32.9)	9 (32.1)	9 (34.6)	0.807
Smoking (*n*, %)	24 (32.9)	6 (21.4)	3 (11.5)	0.122
Heart failure (*n*, %)	6 (8.2)	2 (7.1)	0 (0)	0.346
Coronary artery disease (*n*, %)	9 (12.3)	9 (32.1)	2 (7.7)	0.019
Peripheral artery disease (*n*, %)	1 (1.4)	2 (7.1)	1 (3.8)	0.221
History of ischemic stroke (*n*, %)	18 (24.7)	3 (10.7)	6 (23.1)	0.254

ESUS: embolic stroke of undetermined source; AF: atrial fibrillation; IQR: interquartile range; ^*∗*^ANOVA or Pearson *χ*^2^ test as appropriate.

**Table 2:** Association between embolic stroke patterns and the diagnosis of embolic stroke of undetermined source at baseline (*n* = 127).

Embolic stroke pattern	ESUS (*n* = 26)	No-ESUS (*n* = 101)	OR (95% CI)	*p* value
Multiple acute infarcts (*n*, %)	11 (42.3)	46 (45.5)	0.88 (0.37–2.10)	0.767
Simultaneous involvement of different circulations (*n*, %)	8 (30.8)	39 (38.6)	0.71 (0.28–1.78)	0.461
Multiple infarcts of different ages (*n*, %)	11 (42.3)	26 (25.7)	2.11 (0.86–5.18)	0.102
Isolated cortical ischemia (*n*, %)	4 (15.4)	11 (10.9)	1.49 (0.43–5.12)	0.529
At least one embolic stroke pattern (*n*, %)	16 (61.5)	51 (50.5)	1.57 (0.65–3.79)	0.317

ESUS: embolic stroke of undetermined source; OR: odds ratio; CI: confidence interval.

**Table 3:** Association between embolic stroke patterns and the short-term diagnosis of atrial fibrillation (new atrial fibrillation on baseline ECG or in 7 d Holter) (*n* = 127).

Embolic stroke pattern	Short-term AF (*n* = 23)	Non-short-term AF (*n* = 104)	OR (95% CI)	*p*-value
Multiple acute infarcts (*n*, %)	9 (39.1)	48 (46.2)	0.75 (0.30–1.89)	0.541
Simultaneous involvement of different circulations (*n*, %)	8 (34.5)	39 (37.5)	0.89 (0.35–2.29)	0.807
Multiple infarcts of different ages (*n*, %)	6 (26.1)	31 (29.8)	0.83 (0.30–2.31)	0.723
Isolated cortical ischemia (*n*, %)	3 (13.0)	12 (11.5)	1.15 (0.30–4.46)	0.840
At least one embolic stroke pattern (*n*, %)	10 (43.5)	57 (54.8)	0.64 (0.26–1.58)	0.327

OR: odds ratio; CI: confidence interval.

**Table 4:** Association between embolic stroke patterns and long-term diagnosis of atrial fibrillation (baseline ECG to 12-month follow-up) (*n* = 127).

Embolic stroke pattern	Long-term AF (*n* = 28)	Non-long-term AF (*n* = 99)	OR (95% CI)	*p* value
Multiple acute infarcts (*n*, %)	11 (39.3)	46 (46.5)	0.75 (0.32–1.75)	0.501
Simultaneous involvement of different circulations (*n*, %)	10 (35.7)	37 (37.4)	0.93 (0.39–2.23)	0.872
Multiple infarcts of different ages (*n*, %)	8 (28.6)	29 (29.3)	0.97 (0.38–2.44)	0.941
Isolated cortical ischemia (*n*, %)	4 (14.3)	11 (11.1)	1.33 (0.39–4.56)	0.647
At least one embolic stroke pattern (*n*, %)	13 (46.4)	54 (54.5)	0.72 (0.31–1.68)	0.448

OR: odds ratio; CI: confidence interval.

## Abbreviations

AF: Atrial fibrillation
ESUS: Embolic stroke of undetermined source
cCT: Cranial computed tomography
cMRT: Cranial magnetic resonance tomography
ECG: Electrocardiography
NOACs: New oral anticoagulants
ISRCTN: International Standard Randomized Controlled Trial Number
TIA: Transient ischemic attack
DWI: Diffusion-weighted imaging
ADC: Apparent diffusion coefficient
FLAIR: Fluid-attenuated inversion recovery
NASCET: North American Symptomatic Carotid Endarterectomy Trial
OR: Odds ratio
CI: Confidence interval
IQR: Interquartile range
ANOVA: Analysis of variance
NIHSS: National Institutes of Health Stroke Scale
CRYSTAL-AF: Cryptogenic Stroke and underlying Atrial Fibrillation
EF: Ejection fraction.
