# Supplementary material for: Association between Embolic Stroke Patterns, ESUS Etiology, and New Diagnosis of Atrial Fibrillation: A Secondary Data Analysis of the Find-AF Trial
Source: Stroke Res Treat. 2017 Apr 27;2017:1391843. doi: 10.1155/2017/1391843 (PMC5425845; doi:10.1155/2017/1391843)
Supplement: Supplementary file 1 — The supplementary material includes: additional information about the ESUS-group (reasons for non-fulfillment of the ESUS-criteria) and findings in the subgroup with MR-imaging (association between embolic stroke patterns, ESUS and AF). [file 1391843.f1.docx]

| Supplementary table 1: Reasons for non-fulfillment of the ESUS-criteria | |  |  |
| --- | --- | --- | --- |
|  |  |  |  |
|  | ESUS-criteria not fulfilled and no new diagnosis of AF (n=73) | ESUS-criteria not fulfilled and new diagnosis of AF (n=28) | p-value* |
| Reason |  |  | 0.943 |
| Lacunar stroke (n, %) | 28 (38.3) | 9 (32.2) | … |
| Stenosis >50% NASCET | 30 (41.1) | 12 (42.8) | … |
| MI < 4 weeks | 1 (1.4) | 0 (0) | … |
| AF on baseline ECG or first 24 h of Holter | 10 (13.7) | 6 (21.4) | … |
| Other cardio-embolic source except AF | 1 (1.4) | 0 (0) | … |
| Rare causes of stroke | 3 (4.1) | 1 (3.6) | … |
| ESUS: Embolic stroke of undetermined source; NASCET: North American Symptomatic Carotid Endarterectomy | | | |
| Trial; MI: Myocardial infarction; AF: Atrial fibrillation; ECG: Electrocardiogram; *Pearson χ² test | | |  |
|  |  |  |  |

| Supplementary table 2: Association between embolic stroke patterns and the diagnosis of Embolic Stroke of Undetermined Source at baseline in patients with magnetic resonance imaging only (n=65) | | | | |
| --- | --- | --- | --- | --- |
|  |  |  |  |  |
| Embolic stroke pattern | ESUS (n=11) | No-ESUS (n=54) | OR (95% CI) | p-value |
| Multiple acute infarcts (n, %) | 5 (45.5) | 26 (48.1) | 0.83 (0.23-3.07) | 0.784 |
| Simultaneous involvement of different circulations (n, %) | 3 (27.3) | 24 (44.4) | 0.44 (0.10-1.84) | 0.259 |
| Multiple infarcts of different ages (n, %) | 6 (54.5) | 14 (25.9) | 3.26 (0.86-12.39) | 0.083 |
| Isolated cortical ischemias (n, %) | 3 (27.3) | 4 (7.4) | 4.50 (0.84-23.99) | 0.078 |
| At least one embolic stroke pattern (n, %) | 7 (63.6) | 27 (50) | 1.62 (0.42-6.21) | 0.481 |
| ESUS: Embolic stroke of undetermined source; OR: Odds ratio; CI: Confidence interval | | |  |  |
|  |  |  |  |  |

| Supplementary table 3: Association between embolic stroke patterns and the short-term diagnosis of atrial fibrillation (new atrial fibrillation on baseline ECG or in 7 d Holter) in patients with magnetic resonance imaging only (n=65) | | | | |
| --- | --- | --- | --- | --- |
|  |  |  |  |  |
| Embolic stroke pattern | short term AF (n=10) | No-short term AF (n=55) | OR (95% CI) | p-value |
| Multiple acute infarcts (n, %) | 3 (30) | 28 (50.9) | 0.38 (0.09-1.64) | 0.196 |
| Simultaneous involvement of different circulations (n, %) | 3 (30) | 24 (43.6) | 0.52 (0.12-2.22) | 0.376 |
| Multiple infarcts of different ages (n, %) | 2 (20) | 18 (32.7) | 0.49 (0.09-2.53) | 0.392 |
| Isolated cortical ischemias (n, %) | 1 (10) | 6 (10.9) | 0.87 (0.09-8.13) | 0.903 |
| At least one embolic stroke pattern (n, %) | 3 (30) | 31 (56.4) | 0.30 (0.07-1.31) | 0.110 |
| AF: atrial fibrillation; OR: Odds ratio; CI: confidence interval | |  |  |  |
|  |  |  |  |  |
